# Electronic Cigarettes: an Overlooked Tool to Alleviate Disparities in Tobacco Use Disorder Among People with Mental Health and Substance Use Disorders

**DOI:** 10.1007/s11606-023-08137-z

**Published:** 2023-03-23

**Authors:** Jacqueline T. Vuong, Isabelle Ruedisueli, Catherine S. Beaudin, Holly R. Middlekauff

**Affiliations:** 1 Department of Medicine, UCLA David Geffen School of Medicine, Los Angeles, CA USA; 2 Division of Cardiology, Department of Medicine, UCLA David Geffen School of Medicine, Los Angeles, CA USA; 3 Department of Physiology, UCLA David Geffen School of Medicine, Los Angeles, CA USA

## Abstract

The remarkable decline in cigarette smoking since 1964 has plateaued; approximately 12.5% of Americans still smoke. People who continue to smoke are largely members of marginalized groups, such as people with behavioral health conditions (BHC), encompassing both mental health and substance use disorders. Certified smoking cessation interventions can increase smoking abstinence in trials in people with BHC, yet smoking rates remain markedly increased, leading to increased mortality from smoking-related diseases, and worsening health disparities. A novel approach tailored to the unique needs, characteristics, and circumstances of people with BHC is mandated. One promising approach, the electronic cigarette, has not been embraced in the USA, likely due to an understandable concern for non-smoking young people among whom electronic cigarettes have been popular. Recent data confirm that electronic cigarette use is declining among young people, yet cigarette smoking is not declining among people with BHC. We propose smoking cessation trials utilizing electronic cigarettes in people with BHC. To this goal, the UK has already begun allowing companies to submit their products for approval as medically licensed electronic cigarettes that can be prescribed as smoking cessation aids. Our proposal is timely, backed by evidence, and aims to save hundreds of thousands of American lives.

## SCOPE OF THE PROBLEM

The remarkable decline in smoking prevalence in the USA, from over 40% in 1964 when the 1st Surgeon General’s Report on Smoking and Health was released, to the current 12.5%, is attributable to many factors, including public health policies and educational campaigns, legislation, and the development of behavioral and pharmacological smoking-cessation interventions.^1,2^ Despite the triumph of this multi-pronged approach, smoking prevalence remains very high, and is not declining, in certain populations—especially those who are members of marginalized groups.^3,4^ Smoking prevalence in people with behavioral health conditions (BHC), encompassing both mental health conditions and substance use disorders (SUD), ranges from 25 to 85%, depending on the subgroup.^1^ This increased smoking prevalence is accompanied by an increase in mortality and morbidity from smoking-related illnesses. Annually, approximately 200,000 of the 520,000 deaths attributed to smoking in the USA occur in people with BHC.^5^ People with severe mental illness die 25 years earlier than the general population, and the majority of these early deaths are smoking-related.^1,5^ Accordingly, if we are serious about alleviating disparities in healthcare, it is critical to probe potential reasons why patients with BHC have been left behind in the largely successful public health quest to eliminate smoking, and then to consider alternative, even non-traditional, strategies to achieve smoking cessation. We propose a central role for electronic cigarettes, which may be particularly suited to overcome specific barriers to smoking cessation in people with BHC, but which have been understudied and underutilized in this role.

## BARRIERS TO SMOKING CESSATION IN PEOPLE LIVING WITH BHC

Several factors have been identified which contribute to the long-term ineffectiveness of currently available smoking cessation strategies in those with BHC.^6^ One important factor is the level of nicotine dependence in people with BHC. People with BHC smoke more heavily and have a greater level of nicotine dependence, compared to the general population.^1,6^ Neuroimaging has shown that people with schizophrenia have a pathological expansion in central neural connectivity that is reduced by nicotine administration in a dose-dependent manner, providing biologic basis for self-medication that may make smoking cessation particularly challenging.^7^ This greater nicotine dependence leads to more severe nicotine withdrawal symptoms. People with BHC have more trouble quitting—and are more likely to relapse—compared to the general population.^8–11^ Another barrier to smoking cessation in people living with BHC is that smoking is seemingly accepted as a social norm.^12^ Although smoking has been banned from US hospitals since 1993—this ban does not include facilities that treat BHC, where smoking is often accepted and permitted.^13,14^ In fact, staff at some facilities admit to smoking with patients “to build rapport” and may even use cigarettes as rewards for good behavior.^13^ The high level of nicotine dependence in people with BHC, coupled with healthcare providers’ beliefs that smoking cessation will exacerbate underlying mental illness and/or interfere with drug abstinence, informs these policies.^6,15,16^

## TRIALS OF SMOKING CESSATION IN PEOPLE LIVING WITH BHC

Nonetheless, when studied in randomized controlled trials (RCTs), pharmacological therapies have been shown to be effective in people with BHC and do not exacerbate mental illness or undermine drug abstinence.^13,16–18^ Prochaska et al.^13^ studied motivational tobacco cessation with nicotine replacement therapy (NRT) compared to usual care in an RCT of 224 patients hospitalized in a locked acute psychiatry unit. The endpoint, verified smoking 7-day point prevalence abstinence, was significantly higher in the intervention compared to the usual care group throughout the 18-month follow-up (20% vs 7.7%, 95% confidence interval [CI] 1.22, 8.14, *p* = 0.018, > 80% retention). Rehospitalization rates for mental illness exacerbation were lower in those in the smoking cessation-intervention group. Wu et al.^17^ performed a systemic review and meta-analysis of varenicline for smoking cessation in people with severe mental illness that included 398 participants enrolled in 8 RCTs. Smoking cessation (risk ratio [RR] 4.33, 95% CI 1.96, 9.56) and smoking reduction (RR 6.39, 95% CI 2.22, 10.56) were significantly greater in the varenicline compared to those in the placebo group, without an increase in adverse psychiatric events. A Cochrane review^16^ of smoking cessation interventions from 34 studies involving 5796 people with SUD, including people in treatment and in recovery, concluded that interventions compared to usual care significantly increased smoking abstinence in all groups at follow-up (ranging from 6 weeks to 18 months), without impacting abstinence rates from other drugs or alcohol.

## THE ROLE OF ELECTRONIC CIGARETTES AS A SMOKING CESSATION TOOL

Thus, available, certified smoking cessation interventions compared to usual care can increase smoking abstinence in RCT in people with BHC, yet despite this effectiveness, its implementation has been less successful. Smoking rates remain markedly elevated in this group, leading to marked health disparities. Citing the outcomes of these trials, should we be truly satisfied with the “success” of our currently available smoking cessation tools? Or do we acknowledge that, despite the availability of effective smoking cessation interventions, smoking rates in this most vulnerable BHC group remain unacceptably high? In addition to renewed efforts to improve implementation of available cessation therapies, we believe a fresh approach tailored to the needs, characteristics, and circumstances of people living with BHC is mandated. The goal for smoking cessation in people with BHC should be the same endgame goal that we hold for the general population—finally and completely eliminating smoking.

One such fresh approach could be electronic cigarettes. Limited data from studies conducted in the general population support the notion that electronic cigarettes help adults quit smoking—and remain abstinent. A Cochrane meta-analysis that included 5 studies and 1447 adults who smoked reported that electronic cigarettes with nicotine helped people stop smoking for at least 6 months and were more effective than NRT, nicotine-free electronic cigarettes, behavioral support alone or usual care.^19^ Electronic cigarette use in the short term was not associated with adverse effects. Although more effective for smoking cessation than NRT, electronic cigarettes have not yet been directly compared non-NRT therapies in RCTs. A large proportion of people randomized to electronic cigarettes continue to use electronic cigarettes in the long term.^20^ In fact, the majority of the approximately 10.9 million American adults who currently use electronic cigarettes are either former smokers who report using electronic cigarettes to quit smoking, or current smokers who are using electronic cigarettes to reduce or quit smoking.^21^ Moreover, individuals with schizophrenia have been shown to have a biologic basis for nicotine self-medication that worsens their dependence.^7^ Given that electronic cigarettes have been shown to pharmacokinetically mimic combustible cigarettes,^22^ the use of electronic cigarettes may be particularly efficacious and promote improved adherence in implementation than existing therapies. Electronic cigarettes have not yet been studied in RCTs in people with BHC who smoke, since previous smoking cessation intervention trials conducted in people with BHC were designed and concluded before the widespread availability of electronic cigarettes.

## EXPERIENCE WITH ELECTRONIC CIGARETTES IN PEOPLE LIVING WITH BHC

Although it did not include electronic cigarettes, the Smoking Cessation Intervention for Severe Mental Illness (SCIMITAR +) was a recently published, large pragmatic RCT trial that compared 12-month self-reported, verified 7-day smoking abstinence in 526 people with severe mental illness living in the community randomized to “bespoke” (tailored) smoking cessation intervention versus usual care.^23^ The bespoke therapy included behavioral support as well as pharmacological therapies. At 6, but not 12, months, smoking abstinence was significantly greater in the bespoke intervention group. Although electronic cigarettes were not trialed, over one third of participants in each group used electronic cigarettes for the purpose of quitting smoking. This high uptake of electronic cigarettes was perceived as an indicator of the appeal of electronic cigarettes in this group. Attitudes and experiences with electronic cigarettes were studied in 332 people who smoked who were undergoing treatment for SUD in a residential program.^24^ In this group with SUD, electronic cigarettes were viewed favorably as smoking cessation aids, and in fact, almost half had used electronic cigarettes to help them stop smoking. Those that had used electronic cigarettes tended to be more educated, younger, more motivated to quit, and more likely to self-report as White.^24^ If electronic cigarettes are to be offered as a smoking cessation intervention, care must be made to ensure that they are provided equitably, so that their introduction does not exacerbate pre-existing disparities in smoking and health outcomes.

## EVIDENCE THAT ELECTRONIC CIGARETTES ARE LESS HARMFUL THAN TOBACCO CIGARETTES IN THE SHORT TERM

What is the evidence that electronic cigarettes are truly less harmful than combusted tobacco cigarettes? Existing data are focused on short-term effects. First of all, levels of toxicants and carcinogens are far lower, or not present, in emissions from electronic cigarettes compared to those from tobacco cigarettes, in which over 7000 constituents, including 70 known carcinogens, have been identified.^25,26^ Further, these compounds are detected at much lower levels—if at all—in people who exclusively use electronic cigarettes compared to tobacco cigarettes.^27^ Additionally, markers for future heart and vascular diseases, such as levels of oxidative stress, inflammation, changes in hemodynamics, measures of vascular health, and thrombogenicity, are improved after acute and/or short-term electronic cigarette use compared to tobacco cigarette smoking.^26,28^ Results from pulmonary studies in people who switch from tobacco cigarettes completely have reported either stabilization or improvement in respiratory performance.^29,30^ Practically speaking, since many people who use electronic cigarettes to quit smoking tend to continue to use them in the long term, they may be more accurately considered as a harm reduction tool.^20,21^ Data above describe acute or short-term effects of electronic cigarettes; and although benefits appear sustained at 6 months of combustible smoking cessation, definite evidence that electronic cigarettes are less harmful must depend on long-term outcome studies—data that do not yet exist.

## ELECTRONIC CIGARETTES FOR SMOKING CESSATION: PERSPECTIVES IN THE USA AND ABROAD

In 2018, the Royal Australian and New Zealand College of Psychiatrists published a position paper supporting legislation and regulation of electronic cigarettes as part of a harm reduction smoking-cessation intervention for people with BHC.^31^ Additionally, in 2019, the National Health Services committed to funding the option of switching to electronic cigarettes in people with BHC who smoke and are long-term users of mental health services.^32^ And in 2021, the UK began accepting applications from electronic cigarette companies for approval of their products as medically licensed electronic cigarettes that can be prescribed as smoking cessation aids.^33^ The development of a medically licensed electronic cigarette would off-set well-founded concerns about variability and quality control. So what are the arguments against including electronic cigarettes as part of a harm reduction strategy in people with BHC? In the USA, enthusiasm for electronic cigarettes has been tempered by the observation that electronic cigarette use among non-smoking middle and high school students skyrocketed in 2019, reaching epidemic proportions, potentially serving as a gateway to a resurgence in tobacco cigarette smoking, or at the very least, leading to another generation addicted to nicotine.^34^ Three years later, the 2022 data show that these concerns were not realized; tobacco cigarette smoking has never been lower among high school students, and electronic cigarette use has also declined by ~ 50%.^35^ Of concern however is that a quarter of current users have become daily users, indicative of nicotine addiction. This must be weighed against the fact that combustible cigarette smoking has not declined among people with BHC, the majority of whom will die from smoking-related diseases.^1^

## SUMMARY

Although not a panacea, electronic cigarettes may represent a powerful harm reduction tool amongst subpopulations traditionally left behind in conventional smoking cessation movements. The argument in favor of studies of electronic cigarettes as a smoking cessation, harm reduction intervention in people with BHC is multi-faceted. People with BHC have higher levels of smoking burdens and nicotine addiction compared to the general population, and they quit at lower rates. Unlike NRT, the nicotine delivery from an electronic cigarette mimics the nicotine pharmacokinetics of tobacco cigarettes unaccompanied by high levels of toxicants and carcinogens.^22^ Thus, electronic cigarettes may be well positioned to satisfy this nicotine addiction, and mitigate the intense nicotine withdrawal symptoms that sabotage many quit attempts. People with BHC want to stop smoking, and indicators suggest that electronic cigarettes would be an acceptable and well-received intervention. Ideally, people with BHC would use electronic cigarettes only as long as necessary to quit smoking completely (no dual use), and then when ready, also quit electronic cigarettes. People with BHC including both mental illness and SUD have frequent interactions with healthcare providers, starting from a young age—providing an opportunity for tailored support for quit attempts. One could envision initiating electronic cigarette protocols within the facilities that treat BHC, paired, finally, with an unconditional ban on combustible tobacco cigarettes. Furthermore, if the implementation of electronic cigarettes for smoking cessation proves to be successful in individuals with BHC, this would encourage further trials in other marginalized communities that are vulnerable to cigarette-associated disparities. We argue that the implementation of a bespoke, multi-pronged approach^36^ that includes electronic cigarettes, as well as certified cessation for people with BHC, parallel to that used in the general population, has the potential to alleviate disparities and improve tobacco-related health outcomes among the nation’s most disadvantaged populations, and saving, literally, hundreds of thousands of lives.^37^
